# Systematic review and meta-analysis of efficacy of mesenchymal stem cells on locomotor recovery in animal models of traumatic brain injury

**DOI:** 10.1186/s13287-015-0034-0

**Published:** 2015-03-26

**Authors:** Weijun Peng, Jing Sun, Chenxia Sheng, Zhe Wang, Yang Wang, Chunhu Zhang, Rong Fan

**Affiliations:** Department of Integrated Chinese and Western Medicine, The Second Xiangya Hospital, Central South University, No.139 Middle Renmin Road, Changsha, Hunan 410011 PR China; Department of Pathology, Development and Regeneration Key Laboratory of Sichuan Province, Chengdu Medical College, Chengdu, Sichuan 610500 PR China; Institute of Integrated Medicine, Xiangya Hospital, Central South University, No. 87 Xiangya Road, Changsha, Hunan 410008 PR China

## Abstract

**Introduction:**

The therapeutic potential of mesenchymal stem cells (MSCs) for traumatic brain injury (TBI) is attractive. Conducting systematic review and meta-analyses based on data from animal studies can be used to inform clinical trial design. To conduct a systematic review and meta-analysis to (i) systematically review the literatures describing the effect of MSCs therapy in animal models of TBI, (ii) determine the estimated effect size of functional locomotor recovery after experimental TBI, and (iii) to provide empirical evidence of biological factors associated with greater efficacy.

**Methods:**

We conducted a systematic search of PubMed, EMBASE, and Web of Science and hand searched related references. Studies were selected if they reported the efficacy of MSCs in animal models of TBI. Two investigators independently assessed the identified studies. We extracted the details of individual study characteristics from each publication, assessed study quality, evaluated the effect sizes of MSCs treatment, and performed stratified meta-analysis and meta-regression, to assess the influence of study design on the estimated effect size. The presence of small effect sizes was investigated using funnel plots and Egger’s tests.

**Results:**

Twenty-eight eligible controlled studies were identified. The study quality was modest. Between-study heterogeneity was large. Meta-analysis showed that MSCs exert statistically significant positive effects on sensorimotor and neurological motor function. For sensorimotor function, maximum effect size in studies with a quality score of 5 was found in the weight-drop impact injury TBI model established in male SD rats, to which syngeneic umbilical cord-derived MSCs intracerebrally at cell dose of (1–5) × 10^6^ was administered r 6 hours following TBI, using ketamine as anesthetic agent. For neurological motor function, effect size was maximum for studies with a quality score of 5, in which the weight-drop impact injury TBI models of the female Wistar rats were adopted, with administration syngeneic bone marrow-derived MSCs intravenously at cell dose of 5 × 10^6^ at 2 months after TBI, using sevofluorane as anesthetic agent.

**Conclusions:**

We conclude that MSCs therapy may improve locomotor recovery after TBI. However, additional well-designed and well-reported animal studies are needed to guide further clinical studies.

**Electronic supplementary material:**

The online version of this article (doi:10.1186/s13287-015-0034-0) contains supplementary material, which is available to authorized users.

## Introduction

Traumatic brain injury (TBI) is the leading cause of long-term disability in children and young adults worldwide [1]. In the USA alone, TBI leaves 80,000 individuals with permanent disabilities and costs more than US$77 billion on average per year [2]. One of the most prevalent and debilitating features in survivors of TBI is motor dysfunction [3]. TBI survivors with motor dysfunctions tend to walk slower, take smaller steps and strides, show greater mediolateral sway, and may step higher to clear obstacles [4]. However, there is currently no effective strategy to treat the functional sequelae associated with TBI, except for palliative treatment or surgery in some cases, as well as neuro-rehabilitation [5].

At present, the beneficial effects of mesenchymal stem cell (MSC)-based therapy for acute neurological injuries in animal models, like TBI [6], have drawn more and more attention. MSCs are multipotent, fibroblast-like cells that were first found in the stromal compartment of bone marrow in the 1970s by Friedenstein and colleagues [7]. In addition to bone marrow, similar populations have been identified in other adult and fetal tissues, such as bone and adipose tissue, skeletal muscle, teeth, pancreas, lung, liver, amniotic fluid, endometrial polyps, menstrual blood, cord blood, and umbilical cord tissues [3,8-11]. In addition to the ability of multilineage cell type differentiation, MSCs have recently been shown to exhibit other coveted properties such as anti-inflammatory, immunomodulatory, anti-apoptotic, trophic, and angiogenic effects [12]. Moreover, MSCs have high potency in the modulation of the body’s immune system [13], the paracrine secretion of multiple growth factors and cytokines [14], and migration to the diseased site of the body [15]. Furthermore, MSCs present relative ease of isolation, efficient *ex vivo* expansion, lack of ethical concerns, and acceptable safety [16-19]. All of these features make MSCs an ideal therapeutic regimen to treat various injuries including stroke [20,21], myocardial infarction [22], acute lung injury [23], and TBI [24]. Several lines of studies have investigated the efficacy of MSCs in TBI patients [25,26]. However, they have mainly focused on issues of safety and feasibility, which was underpowered due to lack of proper randomized control. By contrast, an increasing number of rodent studies have investigated the efficacy of MSCs on neurological recovery, a relevant clinical outcome that is considered pivotal among TBI patients [27-29].

Although MSCs have been acknowledged as a desirable candidate in treating TBI-associated neurological deficits in animal models, further clinical trials are needed. In this regard, systematic reviews and meta-analyses on animal studies can allow such decisions to be made based on the entirety of existing evidence that is synthesized in an unbiased manner [30]. Importantly, systematic reviews and meta-analyses of animal studies can offer a sensible and rational approach to assess the translational potential of promising experimental interventions before decisions are made to proceed with clinical trials. However, to our knowledge, a systemic review and meta-analysis of the therapeutic efficacy of MSCs on TBI has not so far been performed in experimental animal studies. Therefore, in this investigation we intended to conduct a systematic review and meta-analysis to determine whether the evidence from animal experiments was in favor of MSCs, in terms of mitigating the neurobehavioral outcome in TBI animal models, and to yield information used to inform animal and clinical studies on MSC intervention in TBI.

## Methods

The study protocol accomplished in advance of any data collection is available online [31]. The methods and statistical evaluation approach are described in greater detail in this study protocol.

### Search strategy

We searched three electronic databases (PubMed, EMBASE, and Web of Science; 30 November 2014) for controlled studies reporting the efficacy of MSCs in an *in vivo* animal model of TBI. The search terms, applied with various Boolean operators, are summarized in Table S1 in Additional file 1 and were kept broad to capture all potentially relevant articles.

Searches of the databases using these search terms were preformed independently by two individuals. The bibliographies of relevant articles were used to identify further relevant publications. Abstracts were independently screened by two reviewers to identify studies meeting our inclusion criteria (see below), with differences resolved by discussion with a third reviewer.

### Eligibility criteria

#### Types of studies

We included controlled comparative studies (randomized, quasi-randomized, and nonrandomized) assessing the efficacy of MSC therapy in preclinical models of TBI. No language, publication date, or publication status restrictions were imposed.

#### Preclinical animal models

Four widely-used TBI animal models (fluid percussion injury, controlled cortical impact injury, weight-drop impact acceleration injury, and blast injury) were enrolled in this study [32], and these models can mimic at least part of the diverse pathophysiological aspects of TBI. Because our proposed future clinical trials will focus on adults with TBI, neonatal animal models of TBI were excluded due to the existence of possible differences in underlying mechanisms and response to a specific treatment between the two groups.

### Interventions

The preclinical intervention group included animals from studies that examine MSC types (xenogeneic, syngeneic, or allogeneic cells from any tissue source). MSCs were defined using minimal criteria set out in the International Society for Cellular Therapy consensus statement [33]. MSCs must be administered during or following the induction of experimental TBI. Experiments using pretreatment of MSCs were excluded since they are clinically relevant for the prevention, but not for the treatment, of human TBI. In order to focus on nonmanipulated MSCs, studies using differentiated MSCs (for example, MSCs that had been differentiated into endothelial cells) or MSCs engineered to overexpress or underexpress particular genes, or studies using a co-treatment with another therapy or cell type were excluded. However, MSCs that had been labeled or transfected with cellular markers intended for tracing and imaging (green fluorescent protein, lacZ, bromodeoxyuridine, superparamagnetic iron oxide particles, and so forth) were included. Administration of MSCs accompanied by co-culture or concomitant injection of other cell types or co-treatment of any adjuvant products (for example, matrices, scaffolding) were excluded. Control interventions consisted of placebo (saline, culture medium, or similar vehicle).

### Neurobehavioral outcome

We included all methods measuring motor function, including sensorimotor function and neurological motor function, such as the modified Neurological Severity Score, Neurological Severity Score, foot fault tests, the Rotarod test, the behavior test, motor function scores, and so forth [34-36], in which a baseline of normal or pre-TBI function could be clearly established. In order to be aggregated in the meta-analysis, outcomes must be presented as neurobehavioral outcomes. Moreover, the exact animal numbers in each group, the mean effect size, and variance of the consequences are supposed to be reported. Disagreements between investigators were resolved by consensus after discussion.

#### Data extraction

The following items were independently extracted by two investigators from each included study: reference details (publication year and name); recipient animal (rat strain and sex); TBI (traumatic model); MSCs (donor species and tissue source); intervention regime (time from TBI to intervention, administration route, and number of injections); type of anesthetic agent; time of outcome assessment; and motor function measures.

We extracted details of individual study characteristics from each publication, and where a single publication reported more than one experiment these data were extracted and treated as independent experiments. When neurobehavioral tests were performed serially, only data for the final time point were extracted.

In cases of missing data, we contacted the authors to request further information, clarification, or missing data. If data were expressed only graphically, numerical values were requested from the authors; if a response was not received, digital ruler software was applied to estimate numerical values from the graphs. If required data were not presented or obtained, then the study was eliminated from the detailed analysis.

#### Methodological quality of studies

The methodological quality of individual studies was assessed based on a checklist modified from the Collaborative Approach to Meta-Analysis and Review of Animal Data from Experimental Studies (CAMARADES) as described previously with minor modification [37,38]. The checklist was comprised of 10 items: (1) peer review publication; (2) presence of randomization; (3) assessment of dose–response relationship; (4) blinded assessment of behavioral outcome; (5) monitoring of physiological parameters temperature; (6) sample size calculation; (7) statement of compliance with regulatory requirements; (8) use of other alternative anesthetics but not ketamine (because of its marked neuroprotective activity); (9) statement of potential conflicts of interest; and (10) use of accurate/suitable/adequate animal models. One point was given for evidence of each quality criterion.

#### Statistical analysis

In line with the *Cochrane Handbook for Systematic Reviews of Interventions* [39], the global estimated effect of MSCs on motor outcome was determined by calculating the standardized mean difference (equal to the difference in mean outcome between groups divided by the standard deviation of outcomes among participants, reported in units of standard deviation) and 95% confidence intervals (CIs) using a random-effects model to avoid heterogeneity [38]. The standardized mean difference is used as a summary statistic in meta-analyses when studies assess the same outcome but measure the outcome in a variety of ways (for example, multiple studies measuring depression but using different psychometric scales). Within-study and between-study variation or heterogeneity was assessed using Cochran’s *Q* statistic [40,41], with a significant *Q* statistic (*P* <0.10) indicating heterogeneity among studies. Heterogeneity was also assessed using the *I*^2^ metric, with higher values denoting a greater degree of heterogeneity (0 to 40%, little heterogeneity; 30 to 60%, moderate heterogeneity; 50 to 90%, substantial heterogeneity; 75 to 100%, considerable heterogeneity). *I*^2^ ≤ 50% indicates acceptable heterogeneity among studies [39]. For studies comparing different doses and/or times of drug administration with a single control group, we compared control group data with pooled data from all experimental groups.

Stratified meta-analysis was used to explore the influence of the potential factors on estimated effect size [42]. Differences in mean effect sizes were assessed partitioning heterogeneity using the *χ*^*2*^ distribution with *n* – 1 degrees of freedom (df). Bonferroni correction was used to adjust significance levels for multiple comparisons:

$$ \mathrm{Declared}\ \mathrm{significance} = 1\ \hbox{--}\ {\left(1\ \hbox{--}\ \mathrm{denoted}\ \mathrm{significance}\right)}^{\left(1\ /\ \mathrm{number}\ \mathrm{of}\ \mathrm{comparisons}\right)} $$

This correction yielded critical *P* values of 0.0024 for sensorimotor function and 0.0057 for neurological motor function [43,44].

Meta-regression analyses were conducted to reveal potential sources of heterogeneity, as described in a previous study [45]. The presence of small effect sizes was investigated using funnel plots and Egger’s tests. For Egger’s tests, *P* <0.10 was considered to indicate the presence of small effect sizes [40].

All statistical analyses were performed using Review Manager (version 5.3) The Nordic Cochrane Centre, The Cochrane Collaboration, Copenhagen, Denmark and Stata software (version 12.1) StataCorp LP, Texas, USA.

## Results

### Study selection

**Figure 1:**
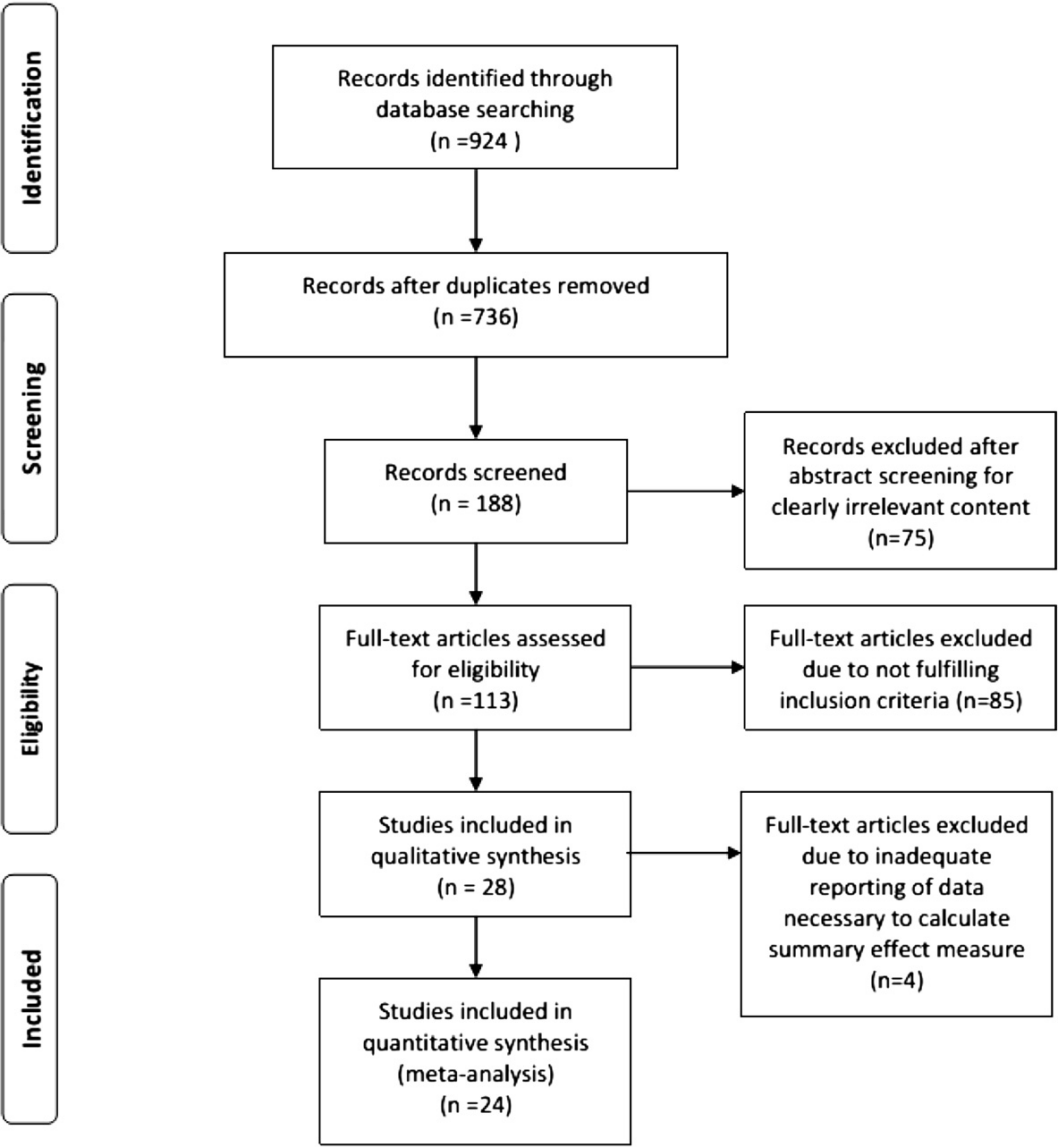
Flow diagram of study search process. A total of 28 studies were identified for systematic review, and the meta-analysis was based on 24 studies only, with four studies excluded due to inadequate reporting of data requisite for calculating the summary effect of measured outcome.

Our review identified 924 publications, of which 28 met our prespecified inclusion criteria [5,6,12,46-70]. Among these, four studies assessing functional neurological were excluded due to inadequate data necessary for calculating the summary effect of measured outcome. Our meta-analysis was thus conducted based on 24 publications, which include 37 comparisons of sensorimotor function and 15 comparisons of neurological motor function (Figure 1).

### Description of studies

The median year of publication was 2011 (range, 2001 to 2014). Bone marrow (22 studies) was the most frequently used MSC tissue source, followed by umbilical cord (four studies). Sprague–Dawley and Wistar rats were the most frequently used rodent strains as recipients, C57Bl/6 mice as the recipient rodent strain was observed in four studies [53,60,62,63], and one study failed to report the recipient rodent strains [50]. Controlled cortical impact injury (17 studies) and weight-drop impact injury (eight studies) were the most frequently used animal models of TBI. The median time interval between TBI induction and MSC intervention was 24 hours, with a longest interval of 2 months. Intravenous injection (19 studies) was the most frequently reported administration route (median total dose, 2 × 10^6^ MSCs; range, 1.5 × 10^5^ to 2 × 10^12^).

The Neurological Severity Score or modified Neurological Severity Score, as the gross neurological deficit score for sensorimotor function, and the Rotarod test were the most frequently used methods to determine neurological motor function (Table S2 in Additional file 1).

### Methodological quality of studies

Overall, the median quality score was 6 (interquartile range, 5 to 7), with scores ranging from 3 to 9. Seven studies did not report randomization of animals into treatment groups, four study assessed dose–response relationships, and 11 studies failed to state that outcome measures were made by experimenters who were blind to animal treatment. Only one study described calculation of the necessary sample size. Seventeen studies stated the compliance with regulatory requirements. Moreover, 13 studies contained a statement of potential conflicts of interest (Table S3 in Additional file 1).

### Overall efficacy

**Figure 2:**
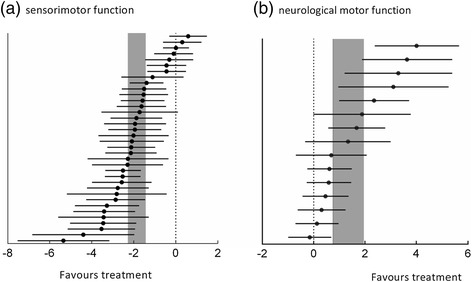
Effects of mesenchymal stem cells on sensorimotor function and neurological motor function. **(a)** Sensorimotor function. **(b)** Neurological motor function. Horizontal lines, mean estimated effect size and 95% confidence interval (CI) for each comparison; vertical gray bars, 95% CI of the pooled estimated effect size.

Efficacy of MSCs on sensorimotor function was evaluated based on measurement of functional outcome in 22 included trials and 37 parallel comparison groups, which involved 657 animals that underwent evaluation for modified Neurological Severity Score, Neurological Severity Score, foot fault tests, elevated body swing test, forelimb akinesia, paw-grasp test, or beam walk tests. The global estimated effect of MSCs was −1.86 (95% CI: −2.27 to −1.44, *P* <0.0001), with significant heterogeneity among studies (χ^2^ = 153.56, df = 36 (*P* <0.0001), *I*^2^ = 77%; Figure 2a). For neurological motor function, in 15 comparisons of nine included studies involving 222 animals – measured using the Rotarod test, the behavior test, and motor function scores – the global estimated effect of MSCs was 1.36 (95% CI: 0.76 to 1.96, *P* <0.0001), with significant heterogeneity among studies (χ^2^ = 50.52, df = 14 (*P* <0.0001), *I*^2^ = 72%; Figure 2b). The pooled analysis indicates that animals in the treatment group showed significantly improved sensorimotor function and neurological motor function compared with animals in the control group (Figure 2a,b).

### Stratified meta-analysis

In stratified meta-analysis, the impact of study quality, type of TBI model, MSC graft type, MSC tissue source, MSC dose, time interval between TBI induction and MSC administration, route of administration, recipient rodents’ sex, recipient rodents’ strain, and anesthetic agent on the effect sizes of sensorimotor and neurological motor function was examined.

**Figure 3:**
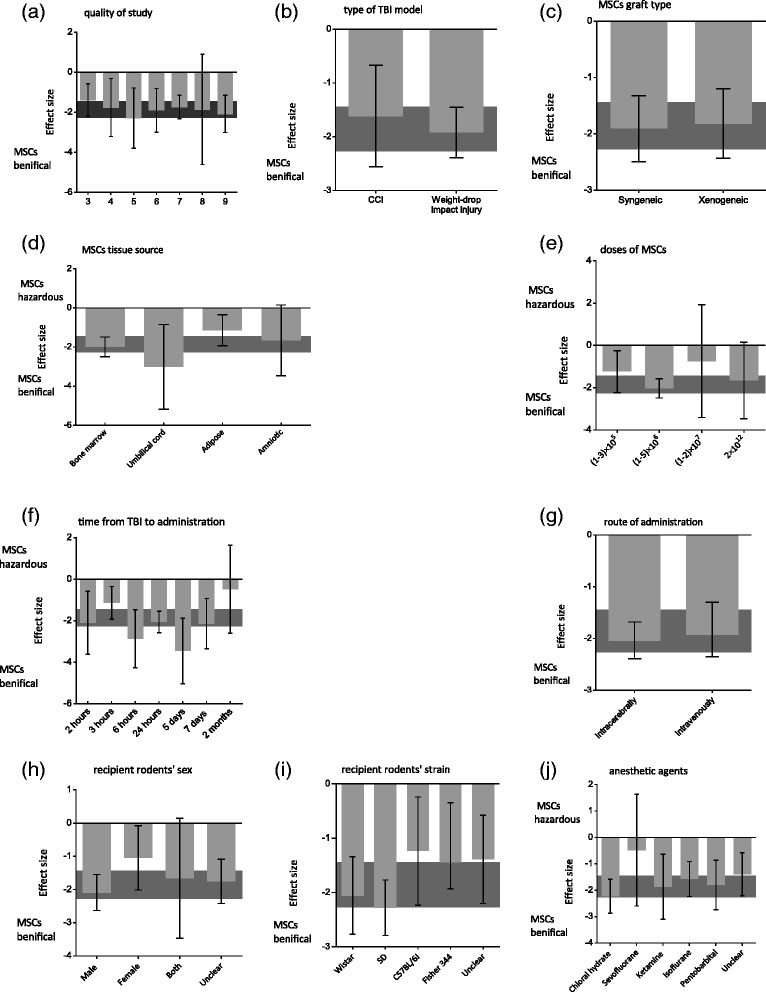
Estimated effect size for sensorimotor function. Estimated effect size for sensorimotor function stratified by **(a)** quality of study, **(b)** type of traumatic brain injury (TBI) model, **(c)** mesenchymal stem cell (MSC) graft type, **(d)** MSC tissue source, **(e)** MSC dose, **(f)** time from TBI to administration, **(g)** administration route, **(h)** recipient rodents’ sex, **(i)** recipient rodents’ strain, and **(j)** anesthetic agents. Grey bands, 95% confidence interval for the global estimated effect size. CCI, controlled cortical impact; SD, Sprague–Dawley.

For sensorimotor function, no significant differences in effect sizes were unveiled relative to the study quality score (χ^2^ = 1.83, df = 6, *P* = 0.93), MSC dose (χ^2^ = 2.75, df = 3, *P* = 0.43), anesthetic agents (χ^2^ = 4.53, df = 5, *P* = 0.48), types of TBI model (χ^2^ = 0.31, df = 1, *P* = 0.58), route of administration (χ^2^ = 0.43, df = 1, *P* = 0.51), time from TBI to intervention (χ^2^ = 11.26, df = 6, *P* = 0.08), MSC donor species (χ^2^ = 1.22, df = 2, *P* = 0.54), recipient rodents’ sex (χ^2^ = 3.47, df = 3, *P* =0.32), MSC graft type (χ^2^ = 0.04, df = 1, *P* = 0.84), MSC tissue source (χ^2^ = 4.48, df = 3, *P* = 0.21), and recipient rodents’ strain (χ^2^ = 8.75, df = 4, *P* = 0.07). However, the effect size was maximum for studies including a quality score of 5 (−2.99, 95% CI: −3.79 to −0.79; Figure 3a), the weight-drop impact injury models (−1.92, 95% CI: − 2.39 to −1.45; Figure 3b), syngeneic grafts (−1.90, 95% CI: −2.49 to −1.32; Figure 3c), umbilical cord-derived MSCs (−3.02, 95% CI: −5.18 to −0.85; Figure 3d), MSCs at a (1 to 5) × 10^6^ cell dose (−2.03, 95% CI: −2.48 to −1.58; Figure 3e), 6 hours following TBI (−2.86, 95% CI: −4.27 to −1.46; Figure 3f), intracerebral administration (−2.04, 95% CI: −2.39 to −1.68; Figure 3g), male rodents (−2.09, 95% CI: −2.63 to −1.55; Figure 3h), Sprague–Dawley rats (−2.28, 95% CI: −2.79 to −1.77; Figure 3i), and ketamine as an anesthetic agent (4.02, 95% CI: 2.38 to 5.66; Figure 3j) (Table S4.1 in Additional file 1).

**Figure 4:**
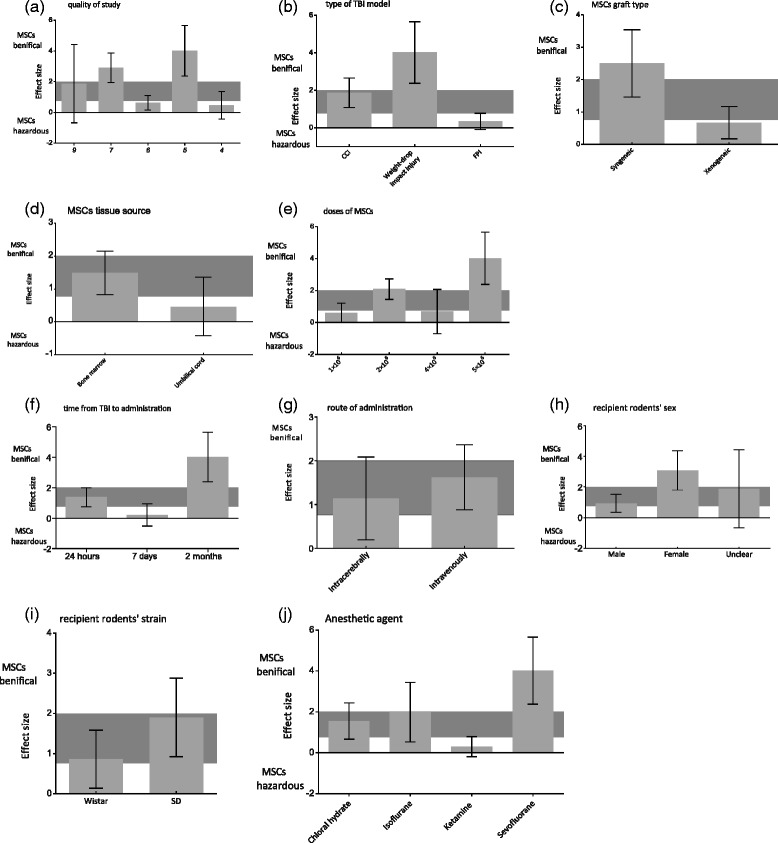
Estimated effect size for neurological motor function. Estimated effect size for neurological motor function stratified by **(a)** quality of study, **(b)** type of traumatic brain injury (TBI) model, **(c)** mesenchymal stem cell (MSC) graft type, **(d)** MSC tissue source, **(e)** MSC dose, **(f)** time from TBI to administration, **(g)** administration route, **(i)** recipient rodents’ sex, **(h)** recipient rodents’ strain, and **(j)** anesthetic agents. Grey bands, 95% confidence interval for the global estimated effect size. CCI, controlled cortical impact; FPI, fluid percussion injury; SD, Sprague–Dawley.

For neurological motor function, significant differences in effect sizes were observed in terms of the study quality score (χ^2^ = 31.91, df = 4, *P* <0.0001), anesthetic agent (χ^2^ = 23.80, df = 3, *P* <0.0001), time from TBI to intervention (χ^2^ = 18.69, df = 2, *P* <0.0001), MSC graft type (χ^2^ = 9.70, df = 1, *P* = 0.002), type of TBI model (χ^2^ = 25.96, df = 2, *P* <0.0001), and MSC dose (χ^2^ = 21.70, df = 3, *P* <0.0001), No significant differences in effect sizes were found relative to route of administration (χ^2^ = 0.61, df = 1, *P* = 0.43), recipient rodents’ sex (χ^2^ = 9.11, df = 2, *P* = 0.01), MSC tissue source (χ^2^ = 3.23, df = 1, *P* = 0.07), and recipient rodents’ strain (χ^2^ = 2.80, df = 1, *P* = 0.09). Moreover, the effect size was maximum for studies with a quality score of 5 (4.02, 95% CI: 2.38 to 5.66; Figure 4a), using the weight-drop impact injury models (4.02, 95% CI: 2.38 to 5.66; Figure 4b), syngeneic grafts (2.50, 95% CI: 1.46 to 3.54; Figure 4c), bone marrow-derived MSCs (1.49, 95% CI: 0.82 to 2.16; Figure 4d), MSCs at 5 × 10^6^ cell dose (4.02, 95% CI: 2.38 to 5.66; Figure 4e), 2 months after TBI (4.02, 95% CI: 2.38 to 5.66; Figure 4f), intravenous administration (1.62, 95% CI: 0.88 to 2.37; Figure 4g), female rodents (3.07, 95% CI: 1.80 to 4.35; Figure 4h), Wistar rats (1.90, 95% CI: 0.92 to 2.88; Figure 4i), and sevofluorane as an anesthetic agent (4.02, 95% CI: 2.38 to 5.66; Figure 4j) (Table S4.2 in Additional file 1).

### Meta-regression analyses

In principle, meta-regression allows the effects of multiple factors to be investigated simultaneously. In order to explore heterogeneity among studies, meta-regression was further conducted. For sensorimotor function, quality of study, type of TBI model, MSC graft type, MSC tissue source, MSC dose, route of administration, and recipient rodents’ sex were the significant sources of heterogeneity for the group (*P* <0.05) (Table S4.1 in Additional file 1). For neurological motor function, quality of study, type of TBI model, MSC graft type, route of administration, recipient rodents’ strain, and anesthetic agent were the significant sources of heterogeneity for the group (*P* <0.05) (Table S4.2 in Additional file 1).

### Publication bias

**Figure 5:**
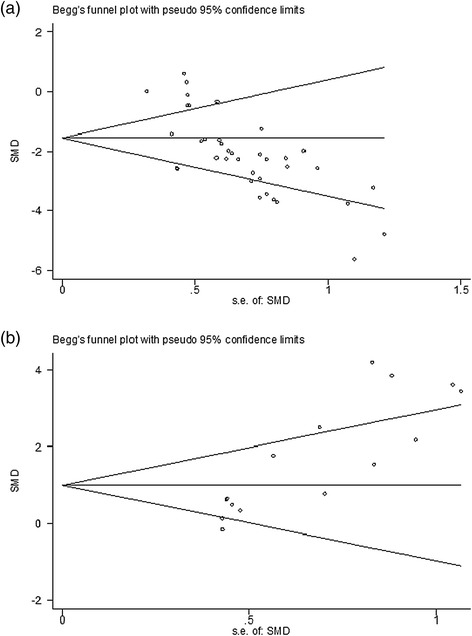
Begg’s funnel plot. **(a)** Sensorimotor function. **(b)** Neurological motor function. There was evidence of small study effects (Egger’s test bias coefficient: −4.9792, 95% confidence interval (CI): −6.729 to −3.229, *P* <0.0001; and 5.6357, 95% CI: 3.6827 to 7.5886, *P* <0.0001). s.e., standard error; SMD, standardized mean difference.

Finally, we sought to identify the presence of small study effects, which may contribute to publication bias. Asymmetry in funnel plots of sensorimotor function indicated the presence of publication bias (Figure 5a; Egger regression, *P* <0.0001). For neurological motor function, asymmetry in funnel plots occurred, which indicated existence of publication bias (Figure 5b; Egger regression, *P* <0.0001).

## Discussion

It has been argued forcefully that it is necessary to conduct systematic reviews and meta-analyses on animal experiments aimed at modeling clinically relevant problems [71-73], since many treatments that lack any evidence of beneficial effect are currently being offered to vulnerable groups of patients [74,75]. Moreover, systematic reviews and meta-analyses of animal experiments not only allow for a more objective appraisal of the research evidence than is allowed by the traditional narrative reviews more commonly associated with animal research, but also offer a sensible and rational approach to assess the translational potential of promising experimental interventions before decisions are made to proceed with clinical trials [76].

The present review is, to our knowledge, the first attempt to systematically collect all available evidence and critically assess and quantify the efficacy of MSCs in animal models of TBI. From the results of our random-effects meta-analysis, we conclude that MSCs might be beneficial in treating experimental TBI in terms of improving sensorimotor and neurological motor function, indicating that MSCs might be a potentially new candidate for neuroprotective activity in the context of TBI. However, this is a meta-analysis of the efficacy of MSCs in animal models of TBI, based on 24 studies only. In addition, the significant statistical heterogeneity and the low quality of many studies included in this review reduce bias for MSCs having a substantial beneficial effect on locomotor recovery in animal models of TBI. Similarly, our previous investigations have shown that statins and erythropoietin were also proven to have neuroprotective properties for animal TBI models, although there was significant statistical heterogeneity and a low quality for many studies included in the previous systematic reviews [30,76].

As anticipated, significant heterogeneity in treatment effect was found between study groups. This finding is typical for systematic reviews in animal studies and validates our choice of a random-effects model, and our summary estimates should be considered as the average efficacy rather than the best estimate of a single true efficacy. The main reasons for heterogeneity in this present review were attributed to the limited number of studies and the small sample sizes within those studies. Another important contribution to this heterogeneity may be the low quality of studies and potential bias of the studies selected for analysis [77]. Moreover, heterogeneity not explained by random error is a consequence of experimental diversity between studies [78].

Next, we performed meta-regression analysis to examine potential sources of heterogeneity. Our results suggested that the effect of MSCs on sensorimotor function and neurological motor function could be associated with some explanatory variables. However, it is important to keep in mind that such analyses are entirely observational by nature and not based on randomized comparisons. Hence, they suffer from the usual limitations of any observational investigation, including bias through confounding by other potential explanatory variables. In addition, we assessed the methodological quality of studies in accordance with previously described standards for preclinical development of neuroprotective drugs with minor modifications [37]. Overall, we found that the quality of the included studies was modest because many failed to report sample size calculation, to report blinded assessment of outcome, or to determine a dose–response relationship, which are important issues generally required in clinical studies [79]. The global estimated effect of MSCs on TBI may therefore be overestimated due to the low quality of studies. This phenomenon is not limited to our investigation; in other systematic reviews of controlled trials in animal models [73,80,81], the statistical heterogeneity between comparisons for all outcomes is also evident, and/or the overall methodological quality of included studies was also poor.

Publication bias is known to be a major problem in the reporting of clinical trials. Here, we showed that publication bias is prevalent in reports of laboratory-based research in animal models of TBI investigating the effect of MSCs on sensorimotor function recovery. Combined with our previous investigations [30,76] and other studies [73,80], it is unlikely that the publication bias reported here is limited to the effect of MSC efficacy on sensorimotor function recovery and is likely to be prevalent in experimental TBI models.

Our study has several limitations, which have also been observed in previous systematic reviews of animal studies [80,82,83]. Firstly, although we had made an extensive effort to identify all relevant studies, only data from published studies were successfully included in our analyses. It is worth mentioning that, at least with regards to motor outcome, there was evidence of a publication bias in favor of studies with large effect sizes. Our analysis did not take unpublished data into account, so the overall effect size might be overstated in our results. Secondly, previous cumulative meta-analyses of *in vivo* data suggested that the effect estimates become stable if approximately 1,500 animals have been included [84]. The current meta-analysis included data from only 879 animals, which limited confidence in effect estimates. Thirdly, we limited our analysis to neurobehavioral outcomes following TBI, largely due to insufficient data regarding histopathology such as the lesion volume. However, functional outcome, in combination with histopathology, may be just as important in terms of assessing potential neuroprotective drugs [22], and this is worthy of further exploration. Fourthly, we present a series of univariate analyses. Multivariate meta-regression or stepwise partitioning of heterogeneity might provide more robust insights, but these techniques have not been well established. Similarly, for continuous variables, the meta-regressions reported here assumed a linear relationship between the independent and dependent variables, which probably represented an oversimplification, at least for some independent variables [85]. Fifthly, extracting multiple pieces of information from a single publication has the potential to introduce bias into systematic reviews because we have observed the experiments of others rather than conducted experiments of our own, and this observational research should be considered as hypothesis-generating only [85]. Lastly, a variety of different metrics were used (for example, pressure, weight, velocity) to evaluate TBI severity, and no studies specified the degree of severity (for example, mild, moderate, or severe). The results of different studies could therefore be more accurately compared if injury severity is reported in a consistent manner. Importantly, although we found that MSC treatment can have beneficial effects in animal models of TBI, the majority of studies used only controlled cortical impact or weight-drop impact injury models. Any animal model may not fully recapitulate all aspects of secondary injury development observed in humans with TBI [32], thereby limiting the extent to which this experimental research translates to a clinical population.

In addition, other types of stem cells have also been used to treat TBI animals, such as neural progenitor cells, neural stem cells, and embryonic stem cells. Improvement of sensorimotor and neurological motor function has also been observed after transplantation of neural stem cells directly to the cortex below the injury cavity or the cortex–hippocampus interface in controlled cortical impact-injured animals, where cells also showed phenotypic evidence of neuronal differentiation [34,86]. Other authors have also shown that transplantation of neural progenitor cells in the acute and chronic post-traumatic period following TBI could enhance motor and cognitive recovery, and promote survival, migration, and neuronal differentiation. Embryonic stem cell transplantation has also demonstrated neurological motor function improvements following lateral fluid percussion brain injury in Sprague–Dawley rats, but may cause tumors, which raises serious safety concerns about the use of such cells in human [87].

As mentioned in our previous systematic reviews [30,76], to improve the transition from animal experiments to human clinical trials, researchers are strongly recommended to consult and follow the Animals in Research: Reporting In Vivo Experiments (ARRIVE) guidelines [88,89] when designing studies and reporting full methodological details to allow others to reproduce and validate their results and also to enable more accurate reviews and meta-analyses. In addition, other short-term outcomes such as lesion volume, brain edema, blood–brain barrier permeability, as well as long-term disability including cognitive, emotional, and behavioral problems should also be examined. Moreover, additional appropriate and standardized TBI models are needed to evaluate the impact of promising pharmacological interventions on TBI.

## Conclusions

Our present investigation suggests that MSCs may have beneficial effect on locomotor recovery in animal models of TBI. The results, however, should be interpreted in light of the known limitations in animal experimental design and methodological quality.

## Additional file

Additional file 1: Table S1. presenting the key search terms used in database searches, **Table S2.** presenting characteristics of the included studies, **Table S3.** presenting the CAMARADES quality item, and **Table S4.** presenting the study characteristics accounting for heterogeneity (**Table S4.1**, sensorimotor function (stratified analysis of stem cell-treated vs. control) and **Table S4.2**, neurological motor function (stratified analysis of MSC-treated vs. control).

## Abbreviations

CI: confidence interval
df: degrees of freedom
MSC: mesenchymal stem cell
TBI: traumatic brain injury
