# Supplementary material for: Systematic review and meta-analysis of efficacy of mesenchymal stem cells on locomotor recovery in animal models of traumatic brain injury
Source: Stem Cell Res Ther. 2015 Mar 26;6(1):47. doi: 10.1186/s13287-015-0034-0 (PMC4425919; doi:10.1186/s13287-015-0034-0)
Supplement: Additional file 1: Table S1. — presenting the key search terms used in database searches, Table S2. presenting characteristics of the included studies, Table S3. presenting the CAMARADES quality item, and Table S4. presenting the study characteristics accounting for heterogeneity (Table S4.1, sensorimotor function (stratified analysis of stem cell-treated vs. control) and Table S4.2, neurological motor function (stratified analysis of MSC-treated vs. control). [file 13287_2015_34_MOESM1_ESM.doc]

**Table S1.**  Key search terms used in database searches

| Traumatic brain injury | Mesenchymal stem cell | | | |
| --- | --- | --- | --- | --- |
| traumatic brain injury | mesenchymal stem cell | | | wharton's jelly |
| traumatic brain injuries | mesenchymal stem cells | | marrow stromal cell | |
| head injury | mesenchymal stromal cell | | | adipose stem cell |
| head injuries | hepatic stem cells |  | | |
| brain injury | muscle-derived satellite cells | | |  |
| brain injuries | umbilical cord stroma stem cell | | |  |
| injury brain | muscle-derived mesenchymal stem cells | | | |
| injuries brain | umbilical cord blood mesenchymal stem cell | | | |
| head trauma | synovial membrane derived mesenchymal stem cells | | | |
| trauma head | periodontal ligament-derived stem cells | | | |

**Table S2**. Characteristics of included studies

| Study | Donor species | Donor tissue | Recipient  rat strain | Injury model | Total animal number | Anesthetic agent | Time from  TBI to intervention | Administration route | Relative dose (Cells) | Neurological and behavior outcomes measurement | Quality Score |
| --- | --- | --- | --- | --- | --- | --- | --- | --- | --- | --- | --- |
| Lu 2001[1] | rat | bone marrow | male Wistar | CCI | 12 | chloral hydrate | 24 hours | intravenously | 2x10^6^ | NSS;the Rotarod test | 6 |
| Mahmood 2001[2] | rat | bone marrow | female Wistar | CCI | 16 | chloral hydrate | 24 hours | intravenously | 2x10^6^ | NSS;the Rotarod test | 6 |
| Lu 2003[3] | rat | bone marrow | male Wistar | CCI | 36 | chloral hydrate | 24 hours | intravenously | 1x10^6^  2x10^6^  4x10^6^ | mNSS;the Corner test scores | 5 |
| Mahmood 2003[4] | human | bone marrow | Male Wistar | CCI | 18 | chloral hydrate | 24 hours | intravenously | 1x10^6^  2x10^6^ | mNSS;the Rotarod test | 6 |
| Hu 2004[5] | rat | bone marrow | Unclear | weight-drop impact | 61 | Unclear | 24 hours | intracerebrally | 2x10^6^ | mNSS | 3 |
| Mahmood 2004[6] | rat | bone marrow | Male/  female Wistar | CCI | 34 | chloral hydrate | 24 hours | intravenously/ intracerebrally | 1x10^6^  2x10^6^ | mNSS;the Rotarod test | 7 |
| Lu 2006[7] | rat | bone marrow | male *SD* | LFPI | 60 | pentobarbital | 72 hours | intravenously | 5x10^6^ | forelimb grip strength test | 3 |
| Qu 2008[8] | mice | bone marrow | female wild type C57BL/ 6J mice | CCI | 12 | chloral hydrate | 24 hours | intravenously | 0.3x10^6^ | the foot fault tests | 6 |
| Wang 2008[9] | Human | Amniotic | Male/  female Wistar | CCI | 80 | chloral hydrate | 24 hours | intravenously/ intracerebrally | 2x10^12^ | NSS | 4 |
| Bonilla 2009[10] | rat | bone marrow | female Wistar | weight-drop impact | 20 | sevofluorane | 2 months | intracerebrally | 5x10^6^ | mNSS; the rotarod test | 5 |
| Harting 2009[11] | rat | bone marrow | SD | CCI | 24 | isoflurane N_2_O/O2 | 24 hours | intravenously | 2x10^6^  4x10^6^ | NSS; the Rotarod test, Balance Beam test, Foot Fault test, | 9 |
| Bakhtiary 2010[12] | rat | bone marrow | male Wistar | CCI | 40 | chloral hydrate | 24 hours | intravenously | 2x10^6^ | mNSS | 7 |
| Kim 2010[13] | human | bone marrow | male  SD | CCI | 110 | isoflurane N_2_O/O2 | 24 hours | intravenously | 2x10^6^ | mNSS and the Rotarod test | 7 |
| Li 2011[14] | human | bone marrow | male Wistar | CCI | 18 | chloral hydrate | 5 days | intravenously | 3x10^6^ | mNSS | 8 |
| Yuan 2011[15] | human | umbilical cord | male Wistar | FPI | 40 | chloral hydrate | 24 hours | intracerebrally | 1.0×10^6^ | Motor function scores | 4 |
| Zanier 2011[16] | human | umbilical  cord blood | male C57Bl/6 mice | CCI | 232 | pentobarbital | 24 hours | intracerebrally | 1.5x10^5^ | neuroscore; beam walk test | 6 |
| Bonilia 2012[17] | rat | bone marrow | Female  Wistar | weigh t-drop impact | 20 | sevofluorane | 2 months | intravenously | 15x10^6^ | mNSS, internal zone Permanence Time (izPT ) | 8 |
| Jiang 2012[18] | rat | bone marrow | Male  SD | CCI | 100 | chloral hydrate | 24 hours | intracerebrally | 1x10^6^ | NSS | 7 |
| Li 2012[19] | human | bone marrow | male Wistar | CCI | 18 | chloral hydrate | 6 hours | intravenously | 3x10^6^ | mNSS | 8 |
| Poltavtseva 2012[20] | human | bone marrow | outbred albino male | weight-drop impact | 33 | chloral hydrate | 24 hours | intravenously | 1x10^6^ | the cylinder test;  limb stimulation test | 5 |
| Zhang2012 [21] | human | umbilical cord | male  *SD* | weight-drop impact | 18 | chloral hydrate | 7 days | intracerebrally | 2×10^7^ | mNSS | 4 |
| Zhao 2012[22] | human | umbilical cord blood | male  *SD* | weight-drop impact | 90 | chloral hydrate | 24 hours | intravenously | 3 × 10^6^ | mNSS | 6 |
| Han 2013[23] | human | bone marrow | male  SD | LFPI | 34 | ketamine &xylazine | Unclear | intracerebrally | 1x10^6^ | The Barnes maze  The rotating rod task test | 6 |
| Zhang 2013[24] | rat | bone marrow | Male  SD | weight-drop impact | 125 | pentobarbital | 2 hours | intravenously | 4x10^6^ | mNSS | 7 |
| Anbari 2014[25] | rat | bone marrow | male Wistar | weight-drop impact | 16 | ketamine &xylazine | 24 hours | intravenously | 3 × 10^6^ | NSS | 5 |
| Pischiutta  [26] | human | bone marrow | Male C57Bl/6 mice | CCI | 170 | pentobarbital | 24 hours | intracerebrally | 1.5× 10^6^ | neuroscore; beam walk |  |
| Tajiri 2014[27] | human | Adipose | male Fisher 344 | CCI | 82 | isoflurane | 3 hours | intravenously | 4x10^6^ | EBST, forelimb akinesia test, and paw-grasp test | 7 |
| Zanier 2014[28] | human | bone marrow | Male C57Bl/6J mice | CCI | Unclear | pentobarbital | 24 hours | intracerebrally | 1.5x10^5^ | neuroscore; beam walk tests | 7 |

Note: CCI：controlled cortical impact; PBS: phosphate-buffered saline; LFPI: lateral fluid percussion injury; Brdu: bromodeoxyuridine; mNSS: modified neurological severity score; EBST: elevated body swing test; DAI: diffuse axonal injury; SD: *Sprague–Dawley*

**Table S3.** CAMARADES Quality Items

| Study | (1) | (2) | (3) | (4) | (5) | (6) | (7) | (8) | (9) | (10) | Quality Score |
| --- | --- | --- | --- | --- | --- | --- | --- | --- | --- | --- | --- |
| Lu 2001[1] | √ | √ |  | √ | √ |  |  | √ |  | √ | 6 |
| Mahmood 2001[2] | √ | √ |  | √ | √ |  |  | √ |  | √ | 6 |
| Lu 2003[3] | √ |  | √ |  |  | √ |  | √ |  | √ | 5 |
| Mahmood 2003[4] | √ |  | √ | √ | √ |  |  | √ |  | √ | 6 |
| Hu 2004[5] | √ |  |  | √ |  |  |  |  |  | √ | 3 |
| Mahmood 2004[6] | √ | √ | √ | √ | √ |  |  | √ |  | √ | 7 |
| Lu 2006[7] | √ |  |  |  |  |  |  | √ |  | √ | 3 |
| Qu 2008[8] | √ | √ |  | √ | √ |  | √ | √ |  | √ | 6 |
| Wang 2008[9] | √ | √ |  |  |  |  |  | √ |  | √ | 4 |
| Bonilla 2009[10] | √ | √ |  |  |  |  | √ | √ | √ | √ | 5 |
| Harting 2009[11] | √ | √ | √ | √ | √ |  | √ | √ | √ | √ | 9 |
| Bakhtiary 2010[12] | √ | √ |  | √ | √ |  | √ | √ |  | √ | 7 |
| Kim 2010[13] | √ | √ |  |  | √ |  | √ | √ | √ | √ | 7 |
| Li 2011[14] | √ | √ |  | √ | √ |  | √ | √ | √ | √ | 8 |
| Yuan 2011[15] | √ | √ |  |  |  |  |  | √ |  | √ | 4 |
| Zanier 2011[16] | √ |  |  |  | √ |  | √ | √ | √ | √ | 6 |
| Bonilla 2012[17] | √ | √ |  | √ | √ |  | √ | √ | √ | √ | 8 |
| Jiang 2012[18] | √ | √ |  | √ |  |  | √ | √ | √ | √ | 7 |
| Li 2012[19] | √ | √ |  | √ | √ |  | √ | √ | √ | √ | 8 |
| Poltavtseva 2012[20] | √ |  |  |  | √ |  | √ | √ |  | √ | 5 |
| Zhang 2012 [21] | √ | √ |  |  |  |  |  | √ |  | √ | 4 |
| Zhao 2012[22] | √ | √ |  | √ |  |  |  | √ | √ | √ | 6 |
| Han 2013[23] | √ | √ |  | √ |  |  | √ |  | √ | √ | 6 |
| Zhang 2013[24] | √ | √ |  |  | √ |  | √ | √ | √ | √ | 7 |
| Anbari 2014[25] | √ | √ |  |  |  |  | √ |  | √ | √ | 5 |
| Tajiri N 2014[27] | √ | √ |  | √ | √ |  | √ | √ |  | √ | 7 |
| Pischiutta[26] | √ |  |  | √ | √ |  | √ | √ | √ | √ | 7 |
| Zanier 2014[28] | √ | √ |  | √ | √ |  | √ | √ |  | √ | 7 |

Note: (1)peer review publication;(2)presence of randomization;(3)assessment of dose-response relationship;(4)blinded assessment of behavioural outcome; (5)monitoring of physiological parameters temperature;(6)sample size calculation;(7) statement of compliance with regulatory requirements;(8) use of anesthetics other than ketamine (because of its marked intrinsic neuroprotective activity);(9)statement of potential conflicts of interest;(10)the use of accurate/suitable/adequate animal models.

**References**

1. Lu D, Mahmood A, Wang L, Li Y, Lu M, Chopp M: **Adult bone marrow stromal cells administered intravenously to rats after traumatic brain injury migrate into brain and improve neurological outcome.** *Neuroreport* 2001, **12:**559-563.

2. Mahmood A, Lu D, Wang L, Li Y, Lu M, Chopp M: **Treatment of traumatic brain injury in female rats with intravenous administration of bone marrow stromal cells.** *Neurosurgery* 2001, **49:**1196-1203; discussion 1203-1194.

3. Lu M, Chen J, Lu D, Yi L, Mahmood A, Chopp M: **Global test statistics for treatment effect of stroke and traumatic brain injury in rats with administration of bone marrow stromal cells.** *J Neurosci Methods* 2003, **128:**183-190.

4. Mahmood A, Lu D, Lu M, Chopp M: **Treatment of traumatic brain injury in adult rats with intravenous administration of human bone marrow stromal cells.** *Neurosurgery* 2003, **53:**697-702; discussion 702-693.

5. Hu DZ, Zhou LF, Zhu JH: **Marrow stromal cells administrated intracisternally to rats after traumatic brain injury migrate into the brain and improve neurological function.** *Chinese Medical Journal* 2004, **117:**1576-1578.

6. Mahmood A, Lu D, Chopp M: **Marrow stromal cell transplantation after traumatic brain injury promotes cellular proliferation within the brain.** *Neurosurgery* 2004, **55:**1185-1192.

7. Lu J, Moochhala S, Moore XL, Ng KC, Tan MH, Lee LK, He B, Wong MC, Ling EA: **Adult bone marrow cells differentiate into neural phenotypes and improve functional recovery in rats following traumatic brain injury.** *Neurosci Lett* 2006, **398:**12-17.

8. Qu C, Mahmood A, Lu D, Goussev A, Xiong Y, Chopp M: **Treatment of traumatic brain injury in mice with marrow stromal cells.** *Brain Res* 2008, **1208:**234-239.

9. Wang GP, Yang B, Guan FX, Du Y, Chang KL, Song LJ, Hu X, Zeng GW: **[Treatment of brain injured rats through transplanting amniotic-derived mesenchymal stem cells in different ways].** *Zhong Nan Da Xue Xue Bao Yi Xue Ban* 2008, **33:**926-930.

10. Bonilla C, Zurita M, Otero L, Aguayo C, Vaquero J: **Delayed intralesional transplantation of bone marrow stromal cells increases endogenous neurogenesis and promotes functional recovery after severe traumatic brain injury.** *Brain injury : [BI]* 2009, **23:**760-769.

11. Harting MT, Jimenez F, Xue H, Fischer UM, Baumgartner J, Dash PK, Cox CS: **Intravenous mesenchymal stem cell therapy for traumatic brain injury.** *J Neurosurg* 2009, **110:**1189-1197.

12. Bakhtiary M, Marzban M, Mehdizadeh M, Joghataei MT, Khoei S, Pirhajati Mahabadi V, Laribi B, Tondar M, Moshkforoush A: **Comparison of transplantation of bone marrow stromal cells (BMSC) and stem cell mobilization by granulocyte colony stimulating factor after traumatic brain injury in rat.** *Iran Biomed J* 2010, **14:**142-149.

13. Kim HJ, Lee JH, Kim SH: **Therapeutic effects of human mesenchymal stem cells on traumatic brain injury in rats: secretion of neurotrophic factors and inhibition of apoptosis.** *J Neurotrauma* 2010, **27:**131-138.

14. Li L, Jiang Q, Qu CS, Ding GL, Li QJ, Wang SY, Lee JH, Lu M, Mahmood A, Chopp M: **Transplantation of marrow stromal cells restores cerebral blood flow and reduces cerebral atrophy in rats with traumatic brain injury: in vivo MRI study.** *J Neurotrauma* 2011, **28:**535-545.

15. Yuan Y, Yang S-y, Zhang J-n: **Human umbilical cord derived mesenchymal stem cell transplantation for rat traumatic brain injury.** *Journal of Clinical Rehabilitative Tissue Engineering Research* 2011, **15:**8424-8428.

16. Zanier ER, Montinaro M, Vigano M, Villa P, Fumagalli S, Pischiutta F, Longhi L, Leoni ML, Rebulla P, Stocchetti N, et al: **Human umbilical cord blood mesenchymal stem cells protect mice brain after trauma.** *Crit Care Med* 2011, **39:**2501-2510.

17. Bonilla C, Zurita M, Otero L, Aguayo C, Rico MA, Rodriguez A, Vaquero J: **Failure of delayed intravenous administration of bone marrow stromal cells after traumatic brain injury.** *J Neurotrauma* 2012, **29:**394-400.

18. Jiang JD, Bu XY, Liu M, Cheng PX: **Transplantation of autologous bone marrow-derived mesenchymal stem cells for traumatic brain injury.** *Neural regeneration research* 2012, **7:**46-53.

19. Li L, Chopp M, Ding GL, Qu CS, Li QJ, Lu M, Wang S, Nejad-Davarani SP, Mahmood A, Jiang Q: **MRI measurement of angiogenesis and the therapeutic effect of acute marrow stromal cell administration on traumatic brain injury.** *Journal of cerebral blood flow and metabolism : official journal of the International Society of Cerebral Blood Flow and Metabolism* 2012, **32:**2023-2032.

20. Poltavtseva RA, Silachev DN, Pavlovich SV, Kesova MI, Yarygin KN, Lupatov AY, Van'ko LV, Shuvalova MP, Sukhikh GT: **Neuroprotective effect of mesenchymal and neural stem and progenitor cells on sensorimotor recovery after brain injury.** *Bulletin of experimental biology and medicine* 2012, **153:**586-590.

21. Zhang R, Qin K, Fa Z, Liu Y, Li P, Cai Y, Jiang X: **[Motor function evaluation in rats receiving umbilical cord mesenchymal stromal cell transplantation for traumatic brain injury using CatWalk automated gait analysis system].** *Nan Fang Yi Ke Da Xue Xue Bao* 2012, **32:**449-455.

22. Zhao JJ, Chen NY, Shen N, Zhao H, Wang DL, Shi J, Wang Y, Cui XF, Yan ZY, Xue H: **Transplantation of human umbilical cord blood mesenchymal stem cells to treat a rat model of traumatic brain injury.** *Neural regeneration research* 2012, **7:**741-748.

23. Han EY, Chun MH, Kim ST, Lim DP: **Injection time-dependent effect of adult human bone marrow stromal cell transplantation in a rat model of severe traumatic brain injury.** *Curr Stem Cell Res Ther* 2013, **8:**172-181.

24. Zhang R, Liu Y, Yan K, Chen L, Chen XR, Li P, Chen FF, Jiang XD: **Anti-inflammatory and immunomodulatory mechanisms of mesenchymal stem cell transplantation in experimental traumatic brain injury.** *J Neuroinflammation* 2013, **10:**106.

25. Anbari F, Khalili MA, Bahrami AR, Khoradmehr A, Sadeghian F, Fesahat F, Nabi A: **Intravenous transplantation of bone marrow mesenchymal stem cells promotes neural regeneration after traumatic brain injury.** *Neural regeneration research* 2014, **9:**919-923.

26. Pischiutta F, D'Amico G, Dander E, Biondi A, Biagi E, Citerio G, De Simoni MG, Zanier ER: **Immunosuppression does not affect human bone marrow mesenchymal stromal cell efficacy after transplantation in traumatized mice brain.** *Neuropharmacology* 2014, **79:**119-126.

27. Tajiri N, Acosta SA, Shahaduzzaman M, Ishikawa H, Shinozuka K, Pabon M, Hernandez-Ontiveros D, Kim DW, Metcalf C, Staples M, et al: **Intravenous transplants of human adipose-derived stem cell protect the brain from traumatic brain injury-induced neurodegeneration and motor and cognitive impairments: cell graft biodistribution and soluble factors in young and aged rats.** *The Journal of neuroscience : the official journal of the Society for Neuroscience* 2014, **34:**313-326.

28. Zanier ER, Pischiutta F, Riganti L, Marchesi F, Turola E, Fumagalli S, Perego C, Parotto E, Vinci P, Veglianese P, et al: **Bone marrow mesenchymal stromal cells drive protective M2 microglia polarization after brain trauma.** *Neurotherapeutics : the journal of the American Society for Experimental NeuroTherapeutics* 2014, **11:**679-695.

**Tables S4. Study characteristics accounting for heterogeneity**

4.1 Sensorimotor function: Stratified analysis of stem cell-treated *vs*. control

|  | Number of studies | Number of Participants | Std. Mean difference  (IV, random,95%CI) | *P** | *P*** |
| --- | --- | --- | --- | --- | --- |
| Pooled estimate | 37 | 657 | -1.86 [-2.27, -1.44] |  |  |
| **Quality of study** |  |  |  |  |  |
| 4 | 4 | 138 | -1.76 [-3.21, -0.32] | 0.93 | 0.033 |
| 5 | 5 | 90 | -2.29 [-3.79, -0.79] |
| 6 | 7 | 88 | -1.90 [-2.99, -0.81] |
| 7 | 13 | 215 | -1.74 [-2.33, -1.16] |
| 8 | 3 | 56 | -1.86 [-4.61, 0.90] |
| 9 | 4 | 40 | -2.09 [-3.01, -1.16] |
| 3 | 1 | 30 | -1.39 [-2.20, -0.58] |
| **Type of TBI model** |  |  |  |  |  |
| Weight-drop | 7 | 128 | -1.62 [-2.56, -0.67] | 0.58 | <0.0001 |
| CCI | 30 | 529 | -1.92 [-2.39, -1.45] |
| **MSC graft types** |  |  |  |
| Syngeneic | 20 | 298 | -1.90 [-2.49, -1.32] | 0.84 | <0.0001 |
| Xenogeneic | 17 | 359 | -1.82 [-2.43, -1.20] |
| **MSCs tissue source** |  |  |  |  |  |
| bone marrow | 26 | 402 | -2.00 [-2.50, -1.49] | 0.21 | 0.022 |
| umbilical cord | 2 | 30 | -3.02 [-5.18, -0.85] |
| Adipose | 6 | 105 | -1.14 [-1.93, -0.35] |
| Amniotic | 3 | 120 | -1.65 [-3.46, 0.15] |
| **Doses of MSCs** |  |  |  |  |  |
| 2*10^12^ | 3 | 120 | -0.74 [-3.41, 1.92] | 0.43 | 0.022 |
| (1-2)*10^7^ | 2 | 38 | -2.03 [-2.48, -1.58] |
| (1-5)*10^6^ | 29 | 459 | -1.23 [-2.23, -0.24] |
| (1-3)*10^5^ | 3 | 40 | -1.65 [-3.46, 0.15] |
| **Time from TBI to administration** | |  |  |  |  |
| 2 months | 2 | 40 | -2.86 [-4.27, -1.46] | 0.08 | 0.102 |
| 7 days | 1 | 18 | -2.06 [-2.58, -1.54] |
| 5 days | 1 | 18 | -3.45 [-5.03, -1.88] |
| 24 hours | 25 | 446 | -2.14 [-3.35, -0.92] |
| 6 hours | 1 | 18 | -1.14 [-1.93, -0.35] |
| 3 hours | 6 | 105 | -0.48 [-2.60, 1.64] |
| 2 hours | 1 | 12 | -2.09 [-3.62, -0.57] |
| **Route of administration** | |  |  |  |  |
| Intracerebrally | 8 | 200 | -2.04 [-2.39, -1.68] | 0.51 | <0.0001 |
| Intravenously | 29 | 457 | -1.83 [-2.35, -1.30] |
| **Recipient rodents’ sex** | |  |  |  |  |
| Male | 23 | 385 | -2.09 [-2.63, -1.55] | 0.32 | 0.023 |
| Female | 6 | 82 | -1.05 [-2.01, -0.08] |
| Unclear | 5 | 70 | -1.75 [-2.42, -1.09] |
| Both | 3 | 120 | -1.65 [-3.46, 0.15] |
| **Recipient rodents’ strain** | | | | | |
| SD | 9 | 118 | -2.28 [-2.79, -1.77] | 0.07 | 0.080 |
| Wistar | 18 | 364 | -2.06 [-2.77, -1.34] |
| Unclear | 1 | 30 | -1.39 [-2.20, -0.58] |
| C57BL/6L | 3 | 40 | -1.23 [-2.23, -0.24] |
| Fisher 344 | 6 | 105 | -1.14 [-1.93, -0.35] |
| **Anesthetic agents** |  |  |  |  |  |
| Ketamine | 1 | 16 | -1.87 [-3.10, -0.64] | 0.48 | 0.256 |
| Sevofluorane | 2 | 40 | -0.48 [-2.60, 1.64] |
| Isoflurane | 11 | 161 | -1.57 [-2.23, -0.91] |
| Chloral hydrate | 20 | 382 | -2.22 [-2.87, -1.58] |
| Pentobarbital | 2 | 28 | -1.80 [-2.74, -0.87] |
| Unclear | 1 | 30 | -1.39 [-2.20, -0.58] |

Note: **P* value for test for subgroup differences. ***P* value for heterogeneity between subgroups with meta-regression analysis.

4.2 Neurological motor function: Stratified analysis of MSCs-treated *vs*. control

|  | Number of studies | Number of Participants | Std. Mean difference  (IV, random,95%CI) | *P** | *P*** |
| --- | --- | --- | --- | --- | --- |
| Pooled estimate | 15 | 222 | 1.39 [0.77, 2.00] |  |  |
| **Quality of study** |  |  |  |  |  |
| 9 | 2 | 20 | 1.87 [-0.67, 4.42] | <0.00001 | 0.003 |
| 7 | 3 | 42 | 2.90 [1.94, 3.86] |
| 6 | 8 | 120 | 0.63 [0.17, 1.09] |
| 5 | 1 | 20 | 4.02 [2.38, 5.66] |
| 4 | 1 | 20 | 0.46 [-0.43, 1.36] |
| **Type of TBI model** |  |  |  |  |  |
| Weight-drop | 1 | 20 | 4.02 [2.38, 5.66] | <0.00001 | 0.003 |
| FPI | 5 | 88 | 0.34 [-0.09, 0.77] |
| CCI | 9 | 114 | 1.86 [1.07, 2.65] |
| **MSC graft types** |  |  |  |  |  |
| Syngeneic | 7 | 82 | 2.50 [1.46, 3.54] | 0.002 | <0.0001 |
| Xenogeneic | 8 | 140 | 0.67 [0.17, 1.17] |
| **MSCs tissue source** |  |  |  |  |  |
| bone marrow | 14 | 202 | 1.49 [0.82, 2.16] | 0.07 | 0.730 |
| umbilical cord | 1 | 20 | 0.46 [-0.43, 1.36] |
| **Doses of MSCs** |  |  |  |  |  |
| 5 *10^6^ | 1 | 20 | 4.02 [2.38, 5.66] | <0.0001 | 0.446 |
| 4 *10^6^ | 1 | 9 | 0.60 [-0.01, 1.21] |
| 2*10^6^ | 6 | 71 | 2.09 [1.44, 2.73] |
| 1*10^6^ | 7 | 122 | 0.69 [-0.69, 2.07] |
| **Time from TBI to administration** | |  |  |  |  |
| 2 month | 1 | 20 | 4.02 [2.38, 5.66] | <0.0001 | 0.778 |
| 7 days | 2 | 30 | 0.21 [-0.52, 0.94] |
| 24hours | 12 | 172 | 1.39 [0.76, 2.01] |
| **Route of administration** | |  |  |  |  |
| Intracerebrally | 7 | 124 | 1.14 [0.20, 2.09] | 0.43 | 0.004 |
| Intravenously | 8 | 98 | 1.62 [0.88, 2.37] |
| **Recipient rodents’ sex** | |  |  |  |  |
| Male | 10 | 164 | 0.93 [0.35, 1.52] | 0.01 | 0.065 |
| Female | 3 | 38 | 3.07 [1.80, 4.35] |
| Unclear | 2 | 20 | 1.87 [-0.67, 4.42] |
| **Recipient rodents’ strain** | |  |  |  |  |
| SD | 7 | 104 | 0.86 [0.14, 1.58] | 0.09 | 0.001 |
| Wistar | 8 | 118 | 1.90 [0.92, 2.88] |
| **Anesthetic agents** |  |  |  |  |  |
| Sevofluorane | 1 | 20 | 4.02 [2.38, 5.66] | <0.0001 | 0.011 |
| Ketamine | 4 | 68 | 0.30 [-0.19, 0.79] |
| Isoflurane | 3 | 36 | 1.99 [0.54, 3.44] |
| Chloral hydrate | 7 | 98 | 1.56 [0.67, 2.44] |

Note: **P* value for test for subgroup differences. ***P* value for heterogeneity between subgroups with meta-regression analysis.
